# Investigating Neophobia Towards New Food Technologies in Italy: The CoNF&TTI Cross-Sectional Study

**DOI:** 10.3390/nu17172825

**Published:** 2025-08-30

**Authors:** Carmela Protano, Federica Valeriani, Patrizia Calella, Giuseppina Caggiano, Annalisa Bargellini, Aida Bianco, Lavinia Bianco, Salvatore Borzì, Anastasia Cataldo, Maria Eugenia Colucci, Laura Dallolio, Chiara de Waure, Gabriella Di Giuseppe, Pasqualina Laganà, Giuseppe La Spada, Francesca Licata, Isabella Marchesi, Alice Masini, Maria Teresa Montagna, Christian Napoli, Stefania Oliva, Giovanna Paduano, Stefania Paduano, Cesira Pasquarella, Concetta Paola Pelullo, Michela Persiani, Vincenzo Romano Spica, Rossella Sacchetti, Giacomo Scaioli, Concetta Arianna Scicchitano, Roberta Siliquini, Francesco Triggiano, Licia Veronesi, Katia Vitale, Francesca Gallè

**Affiliations:** 1 Department of Public Health and Infectious Diseases, Sapienza University of Rome, 00185 Rome, Italy; carmela.protano@uniroma1.it (C.P.); salvatore.borzi@uniroma1.it (S.B.); katia.vitale@uniroma1.it (K.V.); 2 Department of Movement, Human, and Health Sciences, University of Rome “Foro Italico”, 00135 Rome, Italy; vincenzo.romanospica@uniroma4.it; 3 Department of Medical, Movement and Wellbeing Sciences, University of Naples “Parthenope”, 80133 Naples, Italy; patrizia.calella@uniparthenope.it (P.C.); concettapaola.pelullo@uniparthenope.it (C.P.P.); francesca.galle@uniparthenope.it (F.G.); 4 Interdisciplinary Department of Medicine, University of Bari Aldo Moro, 70124 Bari, Italy; giuseppina.caggiano@uniba.it (G.C.); mariateresa.montagna@uniba.it (M.T.M.); francesco.triggiano@uniba.it (F.T.); 5 Department of Biomedical, Metabolic and Neural Sciences, University of Modena and Reggio Emilia, 41125 Modena, Italy; annalisa.bargellini@unimore.it (A.B.); isabella.marchesi@unimore.it (I.M.); stefania.paduano@unimore.it (S.P.); 6 Department of Medical and Surgical Sciences, University of Catanzaro “Magna Græcia”, 88100 Catanzaro, Italy; a.bianco@unicz.it (A.B.); a.scicchitano@unicz.it (C.A.S.); 7 Department of Medical Surgical Sciences and Translational Medicine, Sapienza University of Rome, 00189 Rome, Italy; lavinia.bianco@uniroma1.it (L.B.); christian.napoli@uniroma1.it (C.N.); stefania.oliva@uniroma1.it (S.O.); 8 National Institute for Health, Migration and Poverty (NIHMP), 00153 Rome, Italy; 9 Department of Public Health Sciences and Pediatrics, University of Turin, 10126 Turin, Italy; anastasia.cataldo@unito.it (A.C.); giacomo.scaioli@unito.it (G.S.); roberta.siliquini@unito.it (R.S.); 10 Department of Medicine and Surgery, University of Parma, 43125 Parma, Italy; mariaeugenia.colucci@unipr.it (M.E.C.); cesiraisabellamaria.pasquarella@unipr.it (C.P.); licia.veronesi@unipr.it (L.V.); 11 Department of Biomedical and Neuromotor Science, Alma Mater Studiorum, University of Bologna, 40126 Bologna, Italy; laura.dallolio@unibo.it (L.D.); michela.persiani3@unibo.it (M.P.); 12 Department of Medicine and Surgery, University of Perugia, 06132 Perugia, Italy; chiara.dewaure@unipg.it; 13 Department of Experimental Medicine, University of Campania “Luigi Vanvitelli” Naples, 80138 Naples, Italy; gabriella.digiuseppe@unicampania.it (G.D.G.); giovanna.paduano@unicampania.it (G.P.); 14 Department of Biomedical and Dental Sciences and Morphofunctional Imaging, University of Messina, 98122 Messina, Italy; plagana@unime.it (P.L.); drlaspadag@gmail.com (G.L.S.); 15 Department of Health Sciences, University of Catanzaro “Magna Græcia”, 88100 Catanzaro, Italy; f.licata@unicz.it; 16 Department of Translational Medicine, Amedeo Avogadro University of Eastern Piedmont, 13100 Novara, Italy; alice.masini@uniupo.it; 17 Department of Education Studies “Giovanni Maria Bertin”, Alma Mater Studiorum, University of Bologna, 40126 Bologna, Italy; rossella.sacchetti@unibo.it; 18 A.O.U. Città della Salute e della Scienza, 10126 Turin, Italy

**Keywords:** novel food, food technology, food neophobia, food technology neophobia, sustainability, environmental attitude, undergraduates

## Abstract

**Background/Objectives**: Food technology neophobia (FTN), defined as the reluctance to accept or consume foods produced with novel or emerging food technologies, represents a psychological barrier to the adoption of sustainable and innovative dietary practices. This cross-sectional study investigated the prevalence of food technology neophobia and its associated factors among Italian university students. **Methods:** A total of 1788 undergraduates from 13 universities completed a validated online questionnaire between February and October 2024. The instrument included the Food Technology Neophobia Scale (FTNS), environmental attitude items, and demographic and dietary questions. **Results**: The mean FTNS score was 51.2 ± 14.0, suggesting moderate levels of neophobia. Multivariate logistic regression identified several factors inversely associated with neophobia: male gender (OR = 0.73, *p* = 0.003), paternal university education (OR = 0.73, *p* = 0.024), studying in Northern Italy (OR = 0.64, *p* < 0.001), dietary supplement use (OR = 0.74, *p* = 0.003), and pro-environmental attitudes (OR = 0.97, *p* < 0.001). Conversely, being a commuter student was associated with increased neophobia (OR = 1.29, *p* = 0.031). **Conclusions**: These findings highlight the influence of socio-demographic, behavioral, and attitudinal factors on the acceptance of new food technologies. Tailored strategies are recommended to address FTN in specific subgroups, particularly among female, commuter, and Southern Italian students, to enhance receptivity to food innovation and support sustainable dietary transitions.

## 1. Introduction

### 1.1. Food Neophobia

Food neophobia (FN) refers to the reluctance to try new foods and has been linked to ancestral adaptive behaviors that allowed individuals to avoid risks related to the consumption of harmful foods [1,2,3]. FN is most common in infancy and old age but is also influenced by several biological, economic, and socio-cultural characteristics [4,5,6]. Although FN might be beneficial in evolutionary terms, in modern environments, FN could decrease the diversity of food intake and thus compromise diet quality [7]. For instance, socio-economic status, education, and gender have all been associated with FN, although findings are not always consistent across studies. Some research suggests that males tend to exhibit higher levels of neophobia [4,6], while other studies reported the opposite trend, with females showing greater reluctance towards novel foods [8]. These discrepancies indicate that the role of gender may be influenced by cultural and contextual factors and require further investigation. In addition, non-tasting is frequently related to sensory characteristics (regarding appearance and flavor) and leads to consumers buying familiar rather than new and possibly unpleasant products as a form of risk aversion to economic losses [9,10]. Beyond psychological reluctance, economic barriers such as higher prices of novel foods or those produced with emerging technologies may also hinder their adoption, particularly among low-income consumers [10].

### 1.2. Food Technology Neophobia

Nevertheless, today global challenges such as overpopulation, the depletion of natural resources, and climate change need radical shifts in dietary habits, such as the consumption of more sustainable food [11]. In fact, the current industrial food production is extremely demanding of natural resources and is unable to feed the estimated 9 billion people expected to live in 2050 on earth [12,13]. In fact, as early as 2009, the Food and Agriculture Organization (FAO) calculated that a 70% rise in food production would be needed in the coming decades to feed the entire world [14]. Of particular concern is the environmental impact of meat, fish, and dairy, which have the greatest impact on land use, water use, and greenhouse gas emissions. Meat, eggs, dairy, and aquaculture production already represent approximately 83% of global agricultural land use and 56–58% of agricultural greenhouse gas emissions (~15% of total anthropogenic emissions), while delivering only 37% of total protein [15]. In this context, a more sustainable use of resources will be demanded for food production in meeting rising expectations of food quality, which will be as good as or better than conventional products [16]. In view of the issues associated with animal protein production, alternative protein sources (e.g., insects as food) have also been identified as promising solutions, with the advantage of using less land and water and producing significantly lower greenhouse gas emissions when compared to traditional livestock production [17]. Novel food technologies can provide a contribution to this shift and allow for new food production that can help save the environment and secure food at the same time [18]. However, despite their potential advantages, the use of nanotechnology, genetic modification, 3D printing, or in vitro growth in food production may not be accepted by certain groups of consumers. This reluctance, known as food technology neophobia (FTN), is conceptually distinct from FN and refers specifically to the rejection of foods produced through novel or emerging technologies [19,20,21,22,23]. FTN is increasingly recognized as a major psychological barrier to the adoption of sustainable food innovations and is most often assessed through the Food Technology Neophobia Scale (FTNS) [18]. Cross-national research indicates that FTN varies substantially across populations. For instance, Wang et al. (2023) reported mean FTN scores of 50.6 in China and 55.0 in New Zealand, highlighting cultural differences in the acceptance of novel technologies [23]. Similarly, the systematic review by Wendt and Weinrich (2023) documented the growing application of the FTN score in consumer studies and underlined the heterogeneity of findings across different contexts [18]. A number of correlates of FTN have been identified in international studies, including sex, parental education, cultural background, and pro-environmental attitudes, the latter typically associated with lower FTN [18,24,25,26,27,28,29,30,31,32,33,34]. Despite these insights, the global evidence base remains limited, and further research is needed to clarify the drivers of FTN in diverse populations, particularly among younger generations who will play a decisive role in shaping future food systems.

Therefore, the aim of the present study was to assess neophobia towards innovative food technologies in a sample of young Italian adults. Furthermore, in order to identify possible correlates of FTN in this population, we aimed at exploring its possible relationships with socio-demographic and behavioral characteristics and with the pro-environmental attitude of participants.

## 2. Materials and Methods

### 2.1. Design and Participants

This cross-sectional study took place between February and October 2024 and included undergraduates attending 13 universities distributed across Northern, Central, and Southern Italy. Considering a total population of 463,885 students enrolled in the Italian universities in the academic year 2023–24, a sample of at least 384 undergraduates would have been required, assuming a 95% confidence level and a 50% response proportion. Information was collected using a structured questionnaire developed and hosted on the EU Survey platform, a European Union–approved tool with robust data security features. The questionnaire was administered through a secure link, which was shared with undergraduates by researchers from each participating university during academic courses, following a brief presentation of the study’s objectives and procedures. Students were also encouraged to share the link with their peers to maximize participation.

The STROBE checklist for cross-sectional studies, together with supporting documentation on variable categorization and statistical analysis, is provided in Supplementary Materials (Table S1). The protocol of the study was approved by the Ethics Committee of the University “Foro Italico” in Rome (approval number 179/2024). The trial strictly adhered to the standards outlined in the Helsinki Declaration, guaranteeing ethical strictness at each phase. Informed consent was obtained digitally, with participants giving explicit consent to the use of all their anonymized data for scientific purposes exclusively. Only adult individuals were invited to participate. The anonymity was fully ensured in accordance with Italian data privacy regulations.

### 2.2. Questionnaire and Covariates

The questionnaire was divided into four major sections: the first intended to reveal the participants’ demographic and behavioral profile, the second focused on participants’ attitudes and concerns regarding novel food technologies, and the third on their pro-environmental attitude.

The first section of the questionnaire was designed to gather detailed demographic and lifestyle information from university students in order to identify possible correlates of food technology neophobia. Therefore, participants were asked to indicate their age and gender, nationality, and university. This latter variable was used to categorize them as studying in Northern, Central, or Southern Italy. Parental education levels were assessed by asking students to specify the highest level of education attained by their mother and father, with response options including secondary education or university degree/postgraduate education. To determine academic background, respondents indicated the degree program they were currently pursuing. The variable “field of study” was obtained by categorizing the answers relating to the participants’ course of study into two categories: medical/health-related field and other field. Residence condition (resident/commuter/off-site with respect to the university) and housing situation (living with birth family or not) were also investigated. A question investigated the presence of chronic diseases, with “no”, “yes”, or “prefer not to answer” as possible responses. In addition, with reference to participants’ behaviors and related characteristics, weight and height were collected to calculate their Body Mass Index (kg/m^2^) and then categorize them as underweight, normal weight, overweight, or obese. Dietary habits were examined by asking participants to report the diet they had been following for at least six months. Considering that Italy belongs to the Mediterranean basin, we aimed at assessing possible differences in food technology neophobia between individuals who adhered to the Mediterranean diet model and those who did not. In addition, participants were asked whether they used dietary supplements, with a binary response of “yes” or “no”. Furthermore, smoking habits (yes/quit in the last year/no), alcohol use (often/occasionally/never), and physical activity (regular or not regular practice) were also investigated. Finally, being engaged in food purchasing/preparation was explored with the following possible responses: “always or frequently”, “sometimes”, or “never”.

The second section of the questionnaire was designed to measure participants’ attitudes and concerns regarding novel food technologies. This section comprised 13 statements derived from the Food Technology Neophobia Scale (FTNS), developed by Cox and Evans [22]. Each statement was rated using a 7-point Likert scale (1 = strongly disagree, 7 = strongly agree), allowing for the assessment of participants’ skepticism or acceptance of new food technologies. A brief description of what can be intended as new food technology has been provided at the beginning of the section. The statements addressed various dimensions of food technology neophobia, including concerns about the health implications, environmental impact, naturalness, necessity, and trust in novel food technologies. To ensure measurement validity, reverse scoring was applied where necessary, and a total score (FTNS score) was calculated for each participant, yielding a theoretical range from 13 to 91, with higher scores indicating greater neophobia toward food technologies. The total score was then dichotomized according to its median value.

The third part of the questionnaire consisted of 12 items aimed at assessing pro-environmental attitude, adapted from a validated composite scale developed in a study by Giacalone et al. [24]. This scale was constructed based on a review by Cruz and Manata [25], which identified existing survey instruments for measuring environmental concern and highlighted issues related to measurement reliability and validity. To mitigate these concerns, Giacalone et al. [24] selected items from multiple well-established scales to ensure clarity, relevance, and consistency in assessing individuals’ environmental attitudes. This section of the questionnaire aimed to evaluate respondents’ personal beliefs regarding human interference in nature, environmental degradation, and policy-related concerns. Specifically, it explored perspectives on human responsibility for environmental damage, the urgency of addressing pollution, and trust or skepticism toward scientific and governmental interventions. Additionally, it assessed emotional reactions to pollution, including frustration, anger, or indifference. Each statement was rated on a 7-point Likert scale, ranging from “strongly disagree” (equal to 1) to “strongly agree” (equal to 7). To ensure accurate interpretation, certain items were reverse-scored, and a total environmental concern score was calculated for each participant, with higher values indicating greater pro-environmental concern.

Incomplete questionnaires or those demonstrating clear evidence of unreliable answers, random patterns, or repeated responses were removed from the final analysis to guarantee data integrity and validity.

### 2.3. Statistical Analysis

The quantitative continuous variables were summarized by mean, standard deviation, median, and interquartile range of values, while the categorical variables were summarized by their absolute and relative frequencies as percentages for each category.

The Kolmogorov–Smirnov test was used to check the normality of numerical data distribution. A univariate analysis was conducted to identify statistically significant differences between participants who showed an FTNS score equal to or higher than the median value and those with a score lower than the median value.

The chi-square test was applied for categorical variables. The Mann–Whitney U-test was used for non-normally distributed numerical variables, and the *t*-test was used for normally distributed numerical variables.

A multivariate analysis was carried out by constructing a multiple regression model to identify potential variables influencing the likelihood of FTN among socio-demographic and health characteristics and dietary habits. Age and gender have been included in all models independently of the significance of their association. For regression analysis, the FTNS score higher or equal to the median value was considered as the outcome variable.

The analysis examined multicollinearity, the occurrence of non-necessary variables applying the likelihood ratio test, as well as model calibration and discrimination to build the final models. The associations were assessed either by calculating the odds ratios (OR) and their corresponding 95% confidence intervals (95% CI). The significance was set at *p* = 0.05. The database management and statistical analyses were performed through the software STATA, version 18 (StataCorp LLC, College Station, TX, USA). 

## 3. Results

In total, 1788 participants who completed the questionnaire were analyzed (response rate 0.38%) (Figure S1). The number of respondents per institution ranged between 0.3 and 0.5% of each local population, ensuring a balanced representation of participants across different universities. The sample had a mean age of about 25 years and a higher proportion of female, Italian, and healthy individuals. High parental educational level, attending a university from the center of Italy and a degree course in fields different from life sciences, and living off-site and without birth family were the less represented categories. As for lifestyles, the majority of the sample did not smoke, occasionally used alcohol, regularly practiced physical activity, did not follow the Mediterranean Diet model, did not use food supplements, and was sometimes engaged in food purchasing/preparation. The mean FTNS value for the total sample was 51.16 ± 13.99 (range 14–91, median value 51, interquartile range 17). A total of 882 participants (49.1% of the sample) showed an FTNS score equal to or higher than the median value. Table 1 shows the characteristics of participants grouped by their level of FTNS (lower or equal/higher than the median value), with the *p*-values resulting from the corresponding univariate analysis. All variables but age and pro-environmental attitude showed a normal distribution and were analyzed through the *t*-test.

The group with the higher score showed higher age, lower level of parents’ education, and higher proportion of students from Southern universities, commuters, and students living with their family; they also reported a more frequent use of dietary supplements, lower use of tobacco and alcohol, and higher engagement in food purchasing and preparation than their counterparts; their environmental attitude was lower than that of the first group. However, in the statistical comparison between FTNS groups, significant differences emerged only for age (*p* = 0.015), parents’ level of education (*p* < 0.001), geographical area (*p* < 0.001), residence status (*p* = 0.001), living situation (*p* = 0.030), use of nutritional supplements (*p* = 0.001), and pro-environmental attitude (*p* < 0.001). These characteristics, which significantly differed between FTNS score groups, were used as independent variables for the multivariate analysis.

In the final regression model, collinearity, calibration, and discrimination were evaluated, while the presence of non-essential variables to the model was assessed through the likelihood ratio test. As shown in Table 2, male gender, having a father with a university degree, studying in Northern Italy, and the use of nutritional supplements were associated with a lower likelihood of food technology neophobia.

Conversely, being a commuter student was linked to a higher risk. A stronger pro-environmental attitude was also significantly associated with reduced neophobic tendency towards new food technologies. 

## 4. Discussion

The present study aimed to assess food technology neophobia (FTN) in a large sample of Italian undergraduates and to identify factors possibly associated with this phenomenon. The first interesting result concerns the recovered mean value of food neophobia levels, equal to 51.2 ± 14.0. Notwithstanding the different populations examined, this finding agrees with those reported by Wang et al. [23] that used the same tool for investigating neophobia towards new food technologies in China and New Zealand. In the former case, the value obtained was 50.6 ± 9.6; in the latter case, 55.0 ± 9.3.

Another interesting result is related to gender differences. Indeed, we discovered that males appeared to be less neophobic than females. A similar result emerges in an Australian study [27]. This association could be related to the fact that women are often more health-conscious and cautious regarding food safety, which may reduce their willingness to experiment with novel foods. However, the scientific literature concerning the correlation between gender and neophobic behavior shows conflicting results. The study by Wang et al., in fact, reported that the average acceptance of new, non-thermal food technologies by male respondents was higher than that of females in New Zealand but lower than that of Chinese females [23].

With regard to the influence of the higher educational level of the father on the absence of FTN, to our knowledge, no study investigated the contribution of this variable on food neophobia. However, a previous study in this field reported that a higher level of education is associated with a lower neophobic attitude towards the consumption of cultivated meat, a specific novel food [28]. For off-farm vegetable products, which represent another type of innovative food technology, a high level of education also is correlated with a lower level of neophobia [22,29,30]. Moreover, students from Northern Italy were found to be less neophobic than those from the South, a trend also reported in previous research [28]. This difference may reflect socio-economic and cultural disparities between regions, which we also verified in other studies aimed at exploring Italian undergraduates’ lifestyles [35,36,37]. Northern areas are generally characterized by higher income and education levels and greater exposure to food innovations, which may contribute to lower FTN. By contrast, Southern regions often show stronger attachment to traditional food practices, larger household size with comparable income, and fewer opportunities to encounter diverse food cultures. These conditions may limit familiarity with innovative products and reduce confidence in foods produced through novel technologies.

Commuter students were found to be more neophobic. This may be explained by their limited time to prepare meals—often relying on food prepared by others—and by a stronger attachment to familiar tastes, which reduces opportunities to experiment with new foods. The same can be said for those who claim to use nutritional supplements. The use of the latter reduces food neophobia, probably because they are already used to consuming substances whose taste is not always conventional.

Considering that certain subgroups—such as females, commuter students, and those from Southern Italy—show higher levels of food neophobia, as also reported in other studies, it is essential to implement targeted strategies aimed at increasing their familiarity with innovative foods and fostering more positive attitudes toward sustainable dietary practices [31,32]. University-based educational campaigns, experiential approaches (e.g., tasting sessions, peer workshops, or digital storytelling), and improved food literacy, particularly among students from lower socio-cultural backgrounds, may enhance openness toward unfamiliar foods. Our study shows that pro-environmental attitude is inversely proportional to food neophobia. In fact, being more sensitive to environmental issues makes one less neophobic towards the consumption of food produced through eco-innovative technologies. This result is in line with those in the literature, in which care for the environment is recognized as a determining factor for the acceptance of innovative foods [33,34,37,38]. Beyond behavioral patterns and demographic influences, the reluctance many individuals express toward emerging food technologies brings to light several ethical concerns. This hesitation often stems not merely from a lack of familiarity, but from deeper apprehensions about the reliability of food regulation systems, the authenticity of so-called “natural” products, and the speed at which these innovations enter everyday life [39,40,41]. Certain segments of the population, such as women, students who commute, or individuals rooted in more conservative cultural contexts, tend to exhibit greater caution toward unfamiliar food practices [38]. If sustainability efforts fail to consider these perspectives, there is a risk of excluding those who feel marginalized or unheard. Encouraging broader acceptance of food innovation will require more than educational campaigns or persuasion strategies. It calls for inclusive and respectful dialog spaces where diverse experiences and concerns are acknowledged. Only through such an engagement can trust be nurtured and meaningful progress in reshaping food systems be achieved [41]. Other than their potential to support sustainability transitions, novel foods could also play a role in enhancing food security in specific vulnerable groups if issues related to cost and accessibility are sufficiently addressed.

The present study has some limitations. First of all, due to its cross-sectional design, it evaluated data collected at a single time point, without the possibility to perform comparisons over time. Secondly, although several universities were included throughout the entire Italian territory, they were selected by convenience, and this undermines the representativeness of the sample. All measures relied on self-reported data, which may be subject to recall and social desirability bias. Moreover, the findings refer to the undergraduate population, and they may not reflect the concerns of the general young adult population. In addition, individuals having an adverse approach towards novel foods could be more reluctant to participate in the research, which is an intrinsic limitation of all the surveys based on voluntary participation. Additionally, the sample was gender-imbalanced (approximately 70% female and 29% male), which may limit the generalizability of gender-related findings and should be considered when interpreting the results. Finally, an additional limitation concerns the categorization of FTN. In this study, FTN scores were dichotomized at the median to obtain balanced group sizes and to facilitate the interpretability of logistic regression models. This choice, however, may have reduced the variability of the original scale. Alternative classification strategies (e.g., cut-offs based on standard deviation or interquartile ranges) could be applied in future research to better capture distributional differences.

Despite these limitations, to our knowledge this is the first study which investigated the prevalence of FTN and its potential predictors in a large sample of undergraduates. The findings are therefore of particular relevance, as they provide a rationale for future longitudinal research and may serve as a basis for the development of tailored health promotion interventions.

## 5. Conclusions

This study contributes to highlighting the high prevalence and correlates of FTN among Italian university students. Our results indicate that FTN is related to demographic (gender, father’s education, area of residence), behavioral (supplement use), and attitudinal (pro-environmentalism) factors. In particular, students with a stronger sense of sustainability-related values were less likely to manifest FTN, indicating that environmental consciousness could be a conducive situation for acknowledging food change.

Policy interventions need to take account of both policy initiatives and the socio-economic determinants of FTN. In addition to regulatory approaches, actions such as education campaigns, experiential strategies (i.e., tastings), and the affordability and availability of new foods may be important to create trust and to stimulate consumers’ acceptance. Indeed, the findings underscore the need to track FTN in younger cohorts, whose opinions will be instrumental in the uptake of sustainable food technologies in the future. Targeted and co-development strategies involving education, awareness, and socio-economic support appear as particularly promising ways of promoting greater openness towards food innovation.

## Figures and Tables

**Table 1:** Differences in characteristics of participants grouped by level of neophobia towards new food technologies. Statistically significant results are highlighted in bold (*p* < 0.05).

Variable		Total Sample (n = 1788)	FTNS * Score Below the Median Value (n = 906)	FTNS Score Equal to or Above the Median Value (n = 882)	*p* Value
Age Mean ± SD	Years	24.8 ± 6.9	24.65 ± 6.39	24.94 ± 7.48	0.015 **
Gender n (%)	Female	1250 (69.9)	624 (68.9)	626 (71.0)	0.075
	Male	518 (29)	267 (29.5)	251 (28.5)	
	Not specified	20 (1.1)	15 (1.6)	5 (0.5)	
Nationality n (%)	Italian	1728 (96.6)	878 (96.9)	850 (96.4)	0.528
	Other	60 (3.4)	28 (3.1)	32 (3.6)	
Mother’s educational level n (%)	Until secondary school	1331 (74.4)	640 (70.6)	691 (78.3)	**<0.001**
	Degree/postgraduate education	457 (25.6)	266 (29.4)	191 (21.7)	
Father’s educational level n (%)	Until secondary school	1449 (81)	699 (77.1)	750 (85.0)	**<0.001**
	Degree/postgraduate education	339 (19)	207 (22.9)	132 (15.0)	
Geographical area n (%)	South	660 (36.9)	291 (32.1)	369 (41.8)	**<0.001**
	Center	256 (14.3)	131 (14.5)	125 (14.2)	
	North	767 (42.9)	428 (47.2)	339 (38.4)	
	Unspecified	105 (5.9)	56 (6.2)	49 (5.6)	
Study Area n (%)	Other than life science	570 (31.9)	282 (31.1)	288 (32.7)	0.488
	Life science	1218 (68.1)	624 (68.9)	594 (67.3)	
Residence condition n (%)	Resident	564 (31.5)	293 (32.3)	271 (30.7)	**0.001**
	Commuter	699 (39.1)	318 (35.1)	381 (43.2)	
	Off-site	525 (29.4)	295 (32.6)	230 (26.1)	
Housing situation n (%)	Living with birth family	1098 (61.4)	534 (58.9)	564 (63.9)	**0.030**
	Other	690 (38.6)	372 (41.1)	318 (36.1)	
Chronic condition n (%)	Yes	191 (11)	106 (12.1)	85 (10.0)	0.162
	No	1538 (89)	771 (87.9)	767 (90)	
BMI n (%)	Underweight	122 (6.8)	64 (7.1)	58 (6.6)	0.545
	Normal weight	1248 (69.8)	626 (69.1)	622 (70.5)	
	Overweight	313 (17.5)	156 (17.2)	157 (17.8)	
	Obese	105 (5.9)	60 (6.6)	45 (5.1)	
Diet regimen n (%)	Other diet	1168 (65.3)	588 (64.9)	580 (65.8)	0.703
	Mediterranean Diet	620 (34.7)	318 (35.1)	302 (34.2)	
Use of dietary supplements n (%)	No	1074 (60)	509 (56.2)	565 (64.1)	**0.001**
	Yes	714 (40)	397 (43.8)	317 (35.9)	
Physical activity n (%)	Not regularly	657 (36.7)	333 (36.8)	324 (36.7)	0.900
	Regularly	1131 (63.3)	573 (63.2)	558 (63.3)	
Smoking n (%)	Yes	558 (31.2)	297 (32.8)	261 (29.6)	0.201
	I quit in the last year	99 (5.5)	54 (6.0)	45 (5.1)	
	No	1131 (63.3)	555 (61.2)	576 (65.3)	
Use of alcohol n (%)	Never	274 (15.4)	126 (13.9)	148 (16.8)	0.183
	Occasionally	1111 (62.1)	566 (62.5)	545 (61.8)	
	Often	403 (22.5)	214 (23.6)	189 (21.4)	
Food purchasing/preparation n (%)	Always or frequently	226 (12.6)	102 (11.3)	124 (14.1)	**0.015**
	Sometimes	883 (49.4)	432 (47.7)	451 (51.1)	
	Never	679 (38)	372 (41)	307 (34.8)	
Pro-environmental attitude Mean ± SD		69.18 ± 10.98	70.99 ± 10.15	67.32 ± 11.49	<0.001 **

* FTNS = Food Technology Neophobia Scale; ** Mann–Whitney U Test.

**Table 2:** Results of the regression analysis performed on the FTN score equal to or higher than the median value as an outcome. Statistically significant results are highlighted in bold (*p* < 0.05).

Variable		Odds Ratio (95% Confidence Intervals)	*p*-Value
Age		1.01 (1.00–1.03)	0.166
Gender	Female (reference)	1.00	
	Male	0.73 (0.59–0.89)	**0.003**
Father’s educational level	Until secondary school (reference)	1.00	
	Degree/postgraduate education	0.73 (0.56–0.96)	**0.024**
Mother’s educational level	Until secondary school (reference)	1.00	
	Degree/postgraduate education	0.81 (0.63–1.03)	0.079
Geographical area	South (reference)	1.00	
	Center	0.78 (0.57–1.06)	0.107
	North	0.64 (0.51–0.80)	**<0.001**
Living condition	Resident (reference)	1.00	
	Commuter	1.29 (1.02–1.63)	**0.031**
	Off-site	0.89 (0.66–1.20)	0.450
Living situation	Living with birth family (reference)	1.00	
	Other	1.04 (0.76–1.41)	0.811
Use of dietary supplements	No (reference)	1.00	
	Yes	0.74 (0.61–0.90)	**0.003**
Food purchasing/preparation	Always or frequently	1.00	
	Sometimes	0.99 (0.73–1.34)	0.932
	Never	0.90 (0.62–1.30)	0.577
Pro-environmental attitude		0.97 (0.96–0.98)	**<0.001**

## Data Availability

The original contributions presented in this study are included in the article/Supplementary Material. Further inquiries can be directed to the corresponding author.

## Supplementary Materials

The following supporting information can be downloaded at https://www.mdpi.com/article/10.3390/nu17172825/s1. Table S1: STROBE statement—checklist of items that should be included in reports of cross-sectional studies. Figure S1: Flow diagram of participants’ enrollment in the CoNF&TTI cross-sectional study. Students from 13 Italian universities were invited via EU-Survey link disseminated during classes and peer sharing (exact number not ascertainable). A total of 1793 questionnaires were accessed, of which 5 were excluded due to unreliability or random response patterns. The final sample included 1788 university students.
