# Supplementary material for: Investigating Neophobia Towards New Food Technologies in Italy: The CoNF&TTI Cross-Sectional Study
Source: Nutrients. 2025 Aug 30;17(17):2825. doi: 10.3390/nu17172825 (PMC12430379; doi:10.3390/nu17172825)
Supplement: Supplementary file 1 [file nutrients-17-02825-s001.zip › Figure S1.pdf]

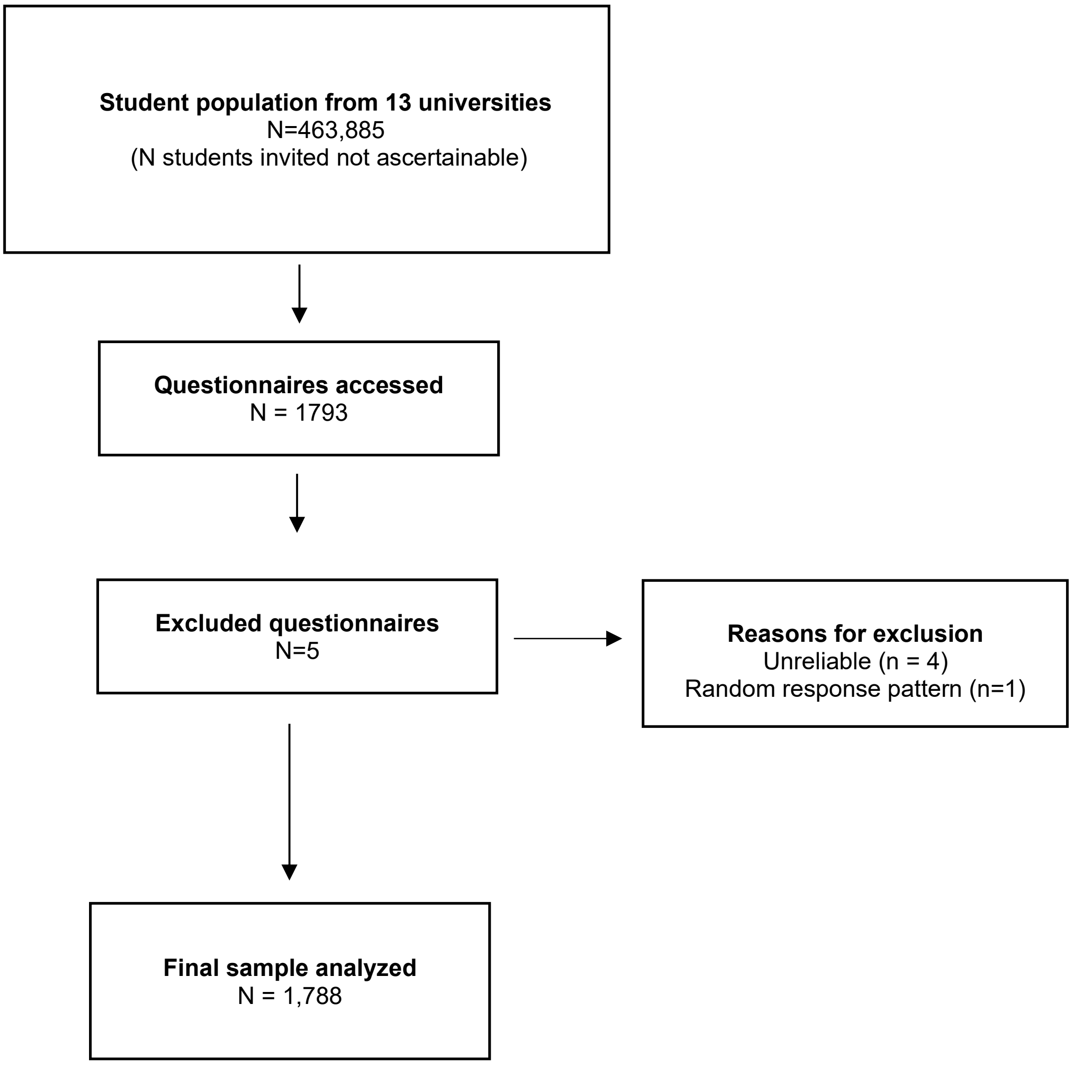

Figure S1. Flow diagram of participants' enrollment in the CoNF&TTI cross-sectional study. Figure S1: Flow diagram of participants' enrollment in the CoNF&TTI cross-sectional study. Students from 13 Italian universities were invited via EU-Survey link disseminated during classes and peer sharing (exact number not ascertainable). A total of 1,793 questionnaires were accessed, of which 5 were excluded due to unreliability or random response patterns. The final sample included 1,788 university students.
